# A novel *DFNB31* mutation associated with Usher type 2 syndrome showing variable degrees of auditory loss in a consanguineous Portuguese family.

**Published:** 2011-06-15

**Authors:** Isabelle Audo, Kinga Bujakowska, Saddek Mohand-Saïd, Sophie Tronche, Marie-Elise Lancelot, Aline Antonio, Aurore Germain, Christine Lonjou, Wassila Carpentier, José-Alain Sahel, Shomi Bhattacharya, Christina Zeitz

**Affiliations:** 1 INSERM, U968, Paris, France; 2 CNRS, UMR_7210, Paris, France; 3 UPMC Univ Paris 06, UMR_S 968, Department of Genetics, Institut de la Vision, Paris, France; 4 Centre Hospitalier National d'Ophtalmologie des Quinze-Vingts, INSERM-DHOS CIC 503,Paris, France; 5 Department of Molecular Genetics, Institute of Ophthalmology, London, UK; 6 Commission Expertise et Evaluation de la SFORL, Paris, France; 7 Plate-forme Post-Génomique P3S, Hôpital Pitié Salpêtrière UPMC, Faculté de Médecine, Paris, France; 8 Fondation Ophtalmologique Adolphe de Rothschild, Paris, France; 9 Department of Cellular Therapy and Regenerative Medicine, Andalusian Molecular Biology and Regenerative Medicine Centre (CABIMER), Seville, Spain

## Abstract

**Purpose:**

To identify the genetic defect of a consanguineous Portuguese family with rod-cone dystrophy and varying degrees of decreased audition.

**Methods:**

A detailed ophthalmic and auditory examination was performed on a Portuguese patient with severe autosomal recessive rod-cone dystrophy. Known genetic defects were excluded by performing autosomal recessive retinitis pigmentosa (arRP) genotyping microarray analysis and by Sanger sequencing of the coding exons and flanking intronic regions of eyes shut homolog–drosophila (*EYS*) and chromosome 2 open reading frame 71 (*C2orf71*). Subsequently, genome-wide homozygosity mapping was performed in DNA samples from available family members using a 700K single nucleotide polymorphism (SNP) microarray. Candidate genes present in the significantly large homozygous regions were screened for mutations using Sanger sequencing.

**Results:**

The largest homozygous region (~11 Mb) in the affected family members was mapped to chromosome 9, which harbors deafness, autosomal recessive 31 (*DFNB31*; a gene previously associated with Usher syndrome). Mutation analysis of *DFNB31* in the index patient identified a novel one-base-pair deletion (c.737delC), which is predicted to lead to a truncated protein (p.Pro246HisfsX13) and co-segregated with the disease in the family. Ophthalmic examination of the index patient and the affected siblings showed severe rod-cone dystrophy. Pure tone audiometry revealed a moderate hearing loss in the index patient, whereas the affected siblings were reported with more profound and early onset hearing impairment.

**Conclusions:**

We report a novel truncating mutation in *DFNB31* associated with severe rod-cone dystrophy and varying degrees of hearing impairment in a consanguineous family of Portuguese origin. This is the second report of *DFNB31* implication in Usher type 2.

## Introduction

Rod-cone dystrophy, also known as retinitis pigmentosa (RP), is a clinically and genetically heterogeneous group of inherited disorders. In most cases, it primarily affects rod function, with night blindness being an initial symptom, followed by secondary cone degeneration leading to progressive visual-field constriction, abnormal color vision, and eventually the loss of central vision. It is the most common inherited form of potentially severe retinal degeneration and blindness, with a frequency of about 1 in 4000 births and more than 1 million affected individuals worldwide [1]. Inheritance of RP can be either autosomal recessive (ar), autosomal dominant (ad) or X-linked (xl) with rare cases of mitochondrial transmission. Recessive and isolated cases account for 50%–60% of all forms of RP [1], with more than 34 implicated genes (RetNet, access March 21st, 2011). Furthermore, arRP can occur with extra-ocular symptoms as in Usher syndrome, in which RP is associated with neurosensory deafness [2]. It is the most common form of syndromic RP and accounts for up to 30% of all RP cases [3]. Three types of Usher syndrome have been clinically described, and are distinguished by variations in the severity and progression of neurosensory deafness, the presence or absence of vestibular abnormalities and the age of onset of the retinal dystrophy [2]. Genetic heterogeneity also characterizes each type and at least nine genes underlying Usher syndrome have been identified [2,4] (Retnet, access March 21st, 2011).

To date, different methods have been used to detect disease-causing mutations underlying heterogeneous disorders such as arRP and Usher syndrome. Direct Sanger sequencing of all known disease-causing genes is labor-intensive, time-consuming and expensive, due to the large genetic heterogeneity and the length of some of the candidate genes, but remains the most precise way to genotype patients. Alternatively, a commercially available genotyping microarray for arRP and Usher syndrome can be applied: this uses the arrayed primer extension (APEX) methodology (Asper Ophthalmics, Tartu, Estonia) [5,6] and allows for the detection of known mutations but fails to discover novel mutations. Another technique uses a custom-designed resequencing chip (customSEQ) to evaluate all exons for which a mutation had previously been reported in 19 genes [7]. Although this technique allows the detection of novel variants, unscreened regions are missed. Next-generation sequencing approaches of candidate genes and/or whole exome or genome sequencing have been initiated; however, data interpretation is complex and further studies are needed before this technology can be used as a daily diagnostic tool. Homozygosity mapping using high-density single nucleotide polymorphism (SNP) microarrays in consanguineous as well as non-consanguineous but geographically isolated families is an efficient tool for localizing genetic defects in homozygous regions and has proven its efficacy in identifying novel arRP genes [8-13].

In the present study, we applied homozygosity mapping to a consanguineous Portuguese family (which was previously excluded for known mutations using the arRP genotyping microarray analysis) to identify the cause of disease.

## Methods

The study protocol adhered to the tenets of the Declaration of Helsinki and was approved by the local Ethics Committee (CPP, Ile de France V). Informed written consent was obtained from each of the study participants.

### Clinical evaluation

The index patient underwent a full ophthalmic examination featuring the assessment of best-corrected visual acuity, kinetic and static perimetry and color vision using 15 desaturated HUE. Full-field and multifocal electroretinography (ERG and mfERG) were performed with Dawson, Trick, Litzkow (DTL) recording electrodes and incorporated the International Society for Clinical Electrophysiology of Vision (ISCEV) recommendations and guidelines (Espion^2^ Diagnosys® for the full field ERG and Veris II for the Multifocal ERG) [14,15]. Clinical assessment was completed with fundus autofluorescence imaging (FAF) and optical coherence tomography (OCT; HRAII® and Spectralis® OCT; Heidelberg Engineering, Dossenheim, Germany). After the genetic results were obtained, a subsequent auditory examination, including pure tone audiometry, was performed on the index patient. Further clinical details for the other affected family members were obtained from their personal health records.

### Molecular genetic analysis

Total genomic DNA was extracted from peripheral blood leucocytes according to the manufacturer’s recommendations (Puregen Kit; Qiagen, Courtaboeuf, France). The DNA sample from the index patient was first analyzed and excluded for all known mutations by applying microarray analysis on a commercially available chip (arRP; ASPER Ophthalmics). Pathogenic variants on eyes shut homolog –drosophila (*EYS*) and chromosome 2 open reading frame 71 (*C2ORF71*) were excluded by directly sequencing the coding exonic and flanking regions of the respective genes [16,17].

DNA samples from the available family members were subsequently genotyped using a 700K genome-wide SNP microarray (Illumina HumanOmniExpress, München, Germany). Genotype calling was done with GenomeStudio software according to the protocols provided by Illumina. In the initial analysis, 686389 SNPs passed the quality control. The homozygous regions were mapped using HomozygosityMapper [18]. Genes located in the mapped regions were sorted using GeneDistiller. The 12 known exons of deafness, autosomal recessive 31 (*DFNB31*) and their flanking regions were PCR amplified in 14 fragments (*DFNB31* RefSeq NM_015404) from the DNA of the index patient and directly sequenced according to the following protocol: PCR reaction, using the oligonucleotide primers indicated in Table 1, was performed from 100 ng of genomic DNA using a commercially available polymerase (HOT FIREPol DNA Polymerase; Solis BioDyne, Tartu, Estonia), 1 mM MgCl_2_ except exon 2, 3, and 10, for which 2.5 mM MgCl_2_ was used, 0.2 mM dNTPs (Solis BioDyne), and buffer B2 (Solis BioDyne) were used at different annealing temperatures as indicated in Table 1. The PCR products were enzymatically purified with 5 µl of a 1:50 dilution (ExoSAP-IT; USB Corporation, Cleveland, OH purchased from GE Healthcare, Orsay, France) at 37 °C for 15 min and the enzyme was deactivated at 80 °C for 15 min. Then, a 0.5 µl sequencing kit (BigDyeTerm v1.1 CycleSeq kit; Applied Biosystems, Courtabœuf, France), 1.25 µl 5× sequencing buffer (BigDye Sequencing buffer; Applied Biosystems) and 0.8 µl 5 µM or 10 µM oligonucleotide were added to the purified PCR products. The cycling conditions were as follows: 95 °C for 20 s, 25 times: 96 °C for 20 s, 50 °C for 10 s, and 60 °C for 2 min. The sequenced product was purified on a presoaked (300 µl H_2_0 for 3 h, centrifuged at 1500× g for 1 min, 150 µl H_2_O centrifuged at 950× g for 5 min) Sephadex G-50 (GE Healthcare) 96-well multiscreen filter plate (Millipore, Molsheim, France) and eluted with an additional 10 µl of H_2_O. The purified product was analyzed on an automated 48-capillary sequencer (ABI 3730 Genetic analyzer, Applied Biosystems) and the results were interpreted using software (SecScape, Applied Biosystems).

**Table 1:** Oligonuleotide primer sequences used for PCR amplification of coding and flanking regions and direct sequencing.

**Amplified fragment**	**Primer sequenced (5′-3′)**	**Annealing temperature**
Exon 1; 1st fragment	GGAACTCGCTGGAAGACTC	58 °C
	GTACAGTGGCTGGATCCTAG	60 °C
Exon 1; 2nd fragment	CTCTTGTGTCTCTCAGGAAC	58 °C
	CACCAGGTCGAAGACGTTG	58 °C
Exon 1; 3rd fragment	GGTTACTGTCTGCCAACGTG	60 °C
	AGACTCTCTCCAAACCACAG	58 °C
Exon 2	CCTTCCTCTAACCTTTGTATC	58 °C
	GAGAATACGGTGTCTGAGAG	58 °C
Exon 3	TCCCTCTAATGTTATCCTCTC	58 °C
	AAAGAGCTCCATGCACAGAG	58 °C
Exon 4	CCAGGTGACTGTATTGTGTG	58 °C
	GTCTGGAGATTGGAGCGAG	58 °C
Exon 5	GCCAACATCTAGAAAGATACC	58 °C
	CTGTGAGGAAGCAGAGAATAC	60 °C
	TCAGCTCTAGAGCAGCTAAG	58 °C
Exon 6	GGAAATGCTGAAGCCAGTTG	58 °C
Exon 7	CCTGTTCCACCTTCTAGTAG	58 °C
	GATTCGAACTCAGGCTGGTC	60 °C
Exon 8	CTCAGCTTCTGTTTGCTCAC	58 °C
	GCTGTCATGGAGAGGAGAG	58 °C
Exon 9	ACTCCTTCTCCGCCTGTTG	58 °C
	CTGGCCTTGCTGTACTCAC	58 °C
Exon 10	TATATAAGCTGCTTCTCTCTTC	58 °C
	ACCTCCCATTCTGTGCCTG	58 °C
Exon 11	GTGGTGTCCTTCCTAATACC	58 °C
	GTAGGGAGAGATGGCAAGTG	60 °C
Exon 12	AAGCCAGGCCTGTCTAACC	58 °C
	GTCCTGCTCTCTTCCTCTC	58 °C

The mutation was described according to the HGVS website. In accordance with this nomenclature, nucleotide numbering reflects cDNA numbering with +1 corresponding to the A of the ATG translation initiation codon in the reference sequence. The initiation codon is codon 1. The correct nomenclature for the mutation was verified by applying Mutalyzer.

## Results

### Molecular genetics

**Figure 1:**
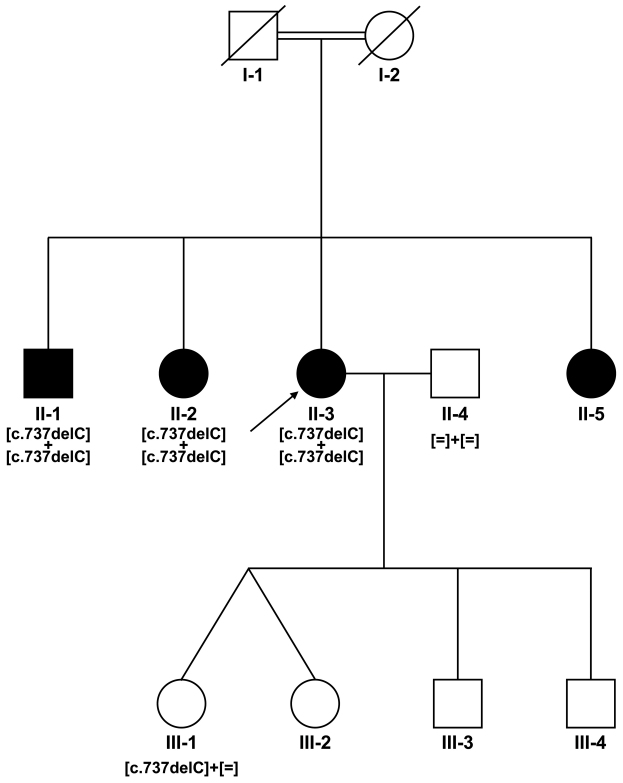
Co-segregation analysis of *DFNB31* mutation in the consanguineous Portuguese family. Filled symbols represent the affected and the unfilled symbols represent the unaffected individuals. Squares depict males and circles depict females. Arrows mark the index patients. Equation symbols represent the unaffected alleles.

**Figure 2:**
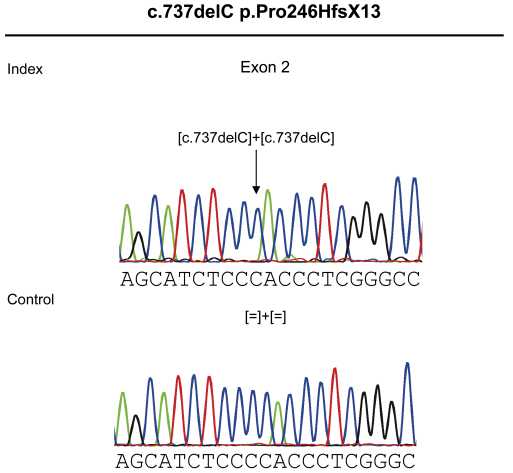
Sequence chromatographs for the novel homozygous mutation c.737delC found in exon 2 of *DFNB31* of the index patient leading to a frameshift and premature stop codon p.Pro246HfsX13 and the respective control sequence below.

The interpretation of the genome-wide SNP microarray-screening results identified four homozygous regions that were only present in the affected subjects, with more than 80% of the maximum score (see pedigree, Figure 1). The largest region was identified on chromosome 9q32-q33.3 and encompassed approximately 11 Mb. The three other homozygous regions were located on chromosome 15q26.1-q26.2, 13q34 and 1p21.3, which revealed lengths of approximately 3, 1.3 and 0.4 Mb, respectively. Within the mapped region on chromosome 9, 102 genes were sorted using GeneDistiller, among which *DFNB31,* encoding whirlin, was the only gene already reported to be involved in rod-cone dystrophy associated with deafness as part of Usher syndrome type 2 (USH2D locus) [19]. No other genes previously reported to underlie retinal dystrophy or deafness were found in either of the homozygous regions. Mutation analysis of *DFNB31* identified a 1-bp deletion, which is predicted to lead to a frameshift resulting in a premature stop codon in exon 2 (c.737delC; p.Pro246HfsX13; Figure 2). Co-segregation analysis on the available samples from the family confirmed the homozygous change in all affected subjects and the heterozygous status of the unaffected daughter of the index patient (Figure 1).

### Clinical characteristics

**Figure 3:**
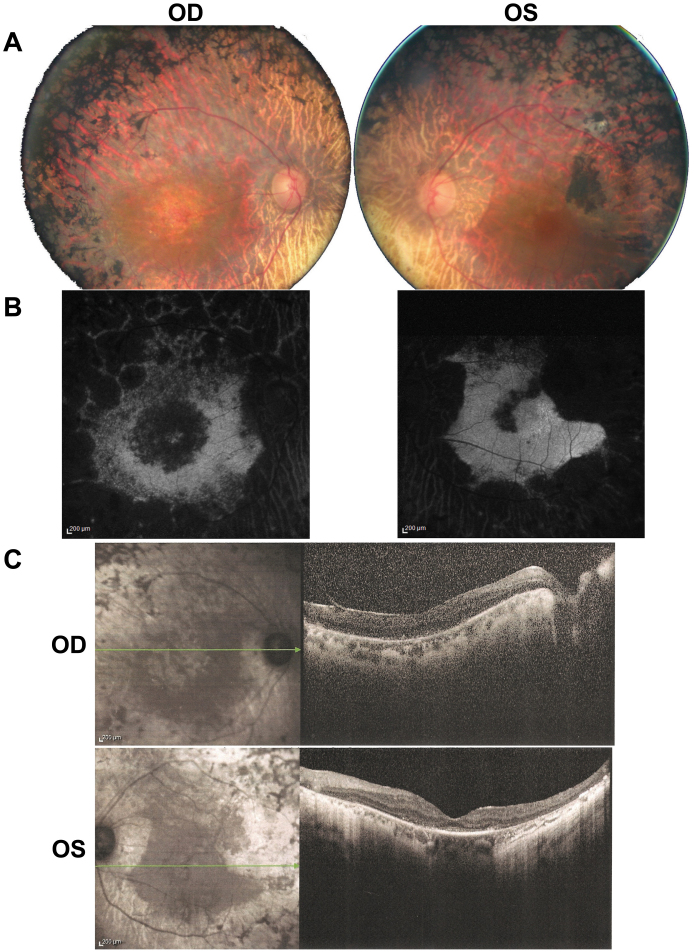
Ophthalmic phenotypic characterization of the right (OD) and left (OS) eye of the index patient. **A**: Color fundus photographs showing widespread changes in the midperiphery associated with atrophic changes in the macular area. **B**: Fundus autofluorescence imaging showing loss of autofluorescence in the midperiphery and in the perifoveal region. **C**: Sprectral domain optical coherence tomography of the right (OD) and left (OS) macula showing perifoveal thinning of the external layer of the retina.

The index patient was 60 years old at the time of the examination. At age 20, she had been diagnosed with RP, causing night vision difficulties, visual field constriction and a mild decrease in visual acuity. There was no other relevant medical history. Ophthalmologic history besides the diagnosis of RP included cataract surgery at age 47 on the right eye and at 54 on the left eye. At the time of the examination, the best-corrected visual acuity of the index patient was reduced to light perception for the right eye and 20/200 for the left eye. Refraction after cataract surgery was, for the right eye +2.5, and for the left eye +2.75. Anterior segment examination was unremarkable, aside from the bilateral artificial intraocular lenses. There was bilateral vitreous detachment and there were few intravitreal cells. Fundus examination revealed pale optic discs, narrow blood vessels, peripheral pigmentary changes with intraretinal pigment migration associated with macular atrophic changes (Figure 3A). Autofluoresence fundus images revealed a loss of autofluorescence outside the vascular arcades as well as loss of autofluorescence in the macular region of the right eye. Similarly, the left eye exhibited loss of autofluorescence in the periphery with better-preserved autofluorescence than the right eye in the macular region (Figure 3B). OCT imaging revealed a widespread disappearance of the outer retinal structures with a relatively better preservation of those in the left eye than in the right (Figure 3C). These findings are consistent with the diagnosis of severe rod-cone dystrophy with macular involvement predominant in the right eye. Binocular Goldman visual field was below 10° with the III4 stimulus. Both full-field and multifocal ERG were undetectable.

**Figure 4:**
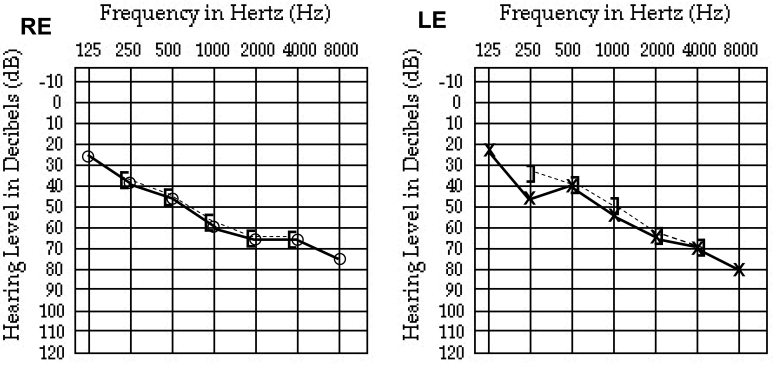
Pure tone audiometry of the right (RE) and left (LE) ears showing bilateral moderately severe hearing loss at −63 dB for the RE and −53 dB for the LE. (**○-)** right and (**x**) left ear air conduction audiometry; (**]- - -)** right and (**[- - - -**) left ear bone conduction audiometry.

In the present study, the index patient did not mention any hearing impairment during the initial medical questionnaire, but her husband subsequently pointed out that she had had more difficulties understanding him for the past few years, suggesting progressive hearing loss. Upon auditory examination, the index patient reported only recent hearing difficulties and bilateral tinnitus. There was no past history of language or developmental abnormalities. There was no reported acute noise or audio-toxic medicine exposure, nor any history of otitis. The patient had no reported balance problems. Her audioscopy was normal, as was her tympanometry. Pure tone audiometry revealed bilateral moderately severe hearing loss at −63 dB for the right ear and −53 dB for the left ear (Figure 4). Hearing aids were subsequently recommended.

The affected brother and sisters were living in Portugal and therefore not available for ophthalmic and auditory examinations. However, they were reported by their respective physicians as having severe visual impairment comparable to that of the index patient and with a similar age of onset. All of the affected siblings notably revealed post-lingual hearing impairment that was more profound than in the index patient. Progressive hearing impairment began before the age of ten and they required hearing aids in their teenage years.

## Discussion

Our study reporting a novel homozygous truncating mutation in *DFNB31* associated with severe rod-cone dystrophy and variable degrees of hearing loss in a consanguineous Portuguese family provides the second report of *DFNB31* involvement in Usher syndrome.

*DFNB31* mutations were initially reported for the mouse ortholog in the whirler mouse (*Whrn*) in autosomal recessive nonsyndromic profound sensoryneural deafness (Deafness, autosomal recessive 31) [20-22] and subsequently in one German family with Usher type 2 syndrome [19].

*DFNB31* encompasses 12 exons and encodes whirlin. Whirlin is a key partner in the Usher protein network, engaged in direct interactions with other Usher proteins [23-27]. These findings suggest that *DFNB31* may play a major role in stereocilia elongation, development and maintenance of cochlear hair cells and in the ciliary transport system between the inner and outer segment of photoreceptors [23,25,27-30]. Alternative splicing of *DFNB31* results in two main whirlin isoforms. The long isoform, encoded by exon 1 to 12, is composed of two PDZ (Postsynaptic density, Disc large, Zo-1) domains at the N-terminus followed by a proline-rich domain and a third PDZ at the C-terminus [21]. The short form, encoded by exon 6 to 12, lacks PDZ_1_ and PDZ_2_ of the N-terminus and only comprises the proline-rich domain and PDZ_3_ [21]. These functional domains are critical in protein–protein interactions within the Usher network. Interestingly, the long isoform is only expressed in photoreceptors within the connecting cilium, whereas both isoforms are expressed in the inner ear [27].

To date, *DFNB31* mutations were identified in only two ar deafness families from large deafness cohort studies [21,22] (Table 2). Similarly, *DFNB31* mutations are rare in Usher syndrome, and Ebberman and coworkers are the only group to report one family with two patients being compound heterozygous for *DFNB31* mutations (Table 2) in 97 USH2 patients [19]. In a recent study, Aller and coworkers [31] documented genetic *DFNB31* variations in a large cohort of 149 Usher type 2, 29 Usher type 1 and six Usher type 3 unrelated patients of European decent, but could not confirm their pathogenic character. Of note, the only *DFNB31* mutations reported in Usher syndrome are located within the coding region specific to the whirlin long isoform. Patients reported by Ebberman and coworkers were compound heterozygous for a nonsense mutation in exon 1, leading to a truncated protein lacking PDZ_1_ and the downstream C-terminal (Table 2) and for one splice site mutation resulting in an in-frame skipping of exon 2 and fusion of PDZ_1_ and PDZ_2_ [19]. Similarly, the novel change we report here (c.737delC; p.Pro246HfsX13) is also located within the whirlin long isoform and is predicted to result in a premature stop codon, which may lead to a truncated protein lacking PDZ_2_ and the rest of the C-terminal part of the long isoform. Alternatively, the mutation could lead to nonsense-mediated decay of the resulting mRNA and reveal a complete loss of the protein [32]. With these mutations affecting exon 1 or 2, the short isoform expression would be unaffected. Conversely, *DFNB31* mutations reported in ar non-syndromic deafness are truncating mutations located in exon 10 [21] and 11 [22], encoding both the long and short isoforms and can result in protein truncation missing both the PDZ_3_ and the downstream C-terminus. Our finding reinforces the phenotype/genotype correlation in *DFNB31* mutations: mutations affecting PDZ_1_ and PDZ_2_, specific of the long isoform, lead to Usher syndrome consistent with a major role of these domains in the retina [27]. Mutations affecting PDZ_3_ present on both isoforms, lead to ar non-syndromic severe deafness, suggesting little, if any, role of this functional domain in photoreceptor function and maintenance. Consequently, one faster screening strategy that could be proposed to investigate *DFNB31* implication in large cohorts of Usher patients would be to sequence only the exons that are specific for coding the long isoform, namely 1 to 5, while studying large cohorts of non-syndromic deafness patients to sequence only exons 6 to 12. However, this screening approach still requires confirmation in ar deafness and Usher cohorts with *DFNB31* mutations.

**Table 2:** Pathogenic variants reported in *DFNB31* with respective phenotype.

**Location in the genomic sequence**	**Codon change**	**Amino acid change**	**Functional consequence**	**phenotype**	**Publications**
Exon 1	c.307C>T	p.Gln103Stop	Protein truncation or NMD affecting PDZ_1_/PDZ_2_ domains	Usher type II	[19]
Exon 2	c.737delC	p.Pro246HisfsX13	Protein truncation or NMD affecting PDZ_1_/PDZ_2_ domains	Usher type II	Present study
Intron 2	c.837+1G>A	Splice mutation in donor site of exon 2	In-frame skipping of exon 2 resulting in PDZ_1_ and PDZ_2_ fusion	Usher type II	[19]
Exon 10	c.2332C>T	p.Arg778Stop	Protein truncation or NMD affecting PDZ_3_	ar deafness	[21]
Exon 11	c.2423delG	p.Gly808AspfsX11	Protein truncation or NMD affecting PDZ_3_	ar deafness	[22]

Ophthalmic examination of the index patient showed severe rod-cone dystrophy with macular involvement. Compared with the two affected siblings (ages 30 and 41 at the time of examination) that were studied by Eberman and coworkers [19], our patient had more severe retinal dystrophy, but her older age (60 at the time of examination) might explain the more advanced disease. In terms of audition, our index patient reported late-onset hearing impairment with moderately severe hearing loss on examination, which was not detected before genetic diagnosis. This suggests that auditory tests are critical while investigating arRP patients and should be encouraged as part of their clinical assessment. The auditory phenotype of our index patient is milder than the one reported by Eberman and coworkers [19]. Although we could not examine the other Portuguese siblings, they reported more severe and early-onset, but post-lingual, deafness. This suggests phenotypic variability in hearing impairment in this family with *DFNB31* mutation. Alternatively, the differences in hearing impairment could be due to additional genetic defects that are distinct from *DFNB31,* which are only carried by the siblings and not by the index patient. In any case, our family and the one reported by Eberman and coworkers [19] differ from the profound pre-lingual hearing impairment reported earlier in ar non-syndromic deafness associated with *DFNB31* mutations disrupting PDZ_3_, thereby underlining the functional importance of this domain in cochlear hair cells [20-22].

Furthermore, our study reinforced the power of homozygosity mapping to identify genetic defects in consanguineous families in an unbiased way. The cost efficiency of this method should be assessed in comparison with systematic candidate gene or mutation microarray screening strategies, but this should be the method of choice for studying cases that deal with consanguineous families or geographically isolated populations in genetically heterogeneous disorders such as retinal dystrophies. Additionally, this strategy increases the chances of recognizing novel phenotype/genotype associations.
